# Caspase-1: A Promising Target for Preserving Blood–Brain Barrier Integrity in Acute Stroke

**DOI:** 10.3389/fnmol.2022.856372

**Published:** 2022-03-18

**Authors:** Xiaodong Ye, Guini Song, Shanshan Huang, Qiming Liang, Yongkang Fang, Lifei Lian, Suiqiang Zhu

**Affiliations:** Department of Neurology, Tongji Hospital, Tongji Medical College, Huazhong University of Science and Technology, Wuhan, China

**Keywords:** caspase-1, ischemic stroke, hemorrhagic stroke, blood-brain barrier, pyroptosis, cerebral edema, hemorrhagic transformation

## Abstract

The blood–brain barrier (BBB) acts as a physical and biochemical barrier that plays a fundamental role in regulating the blood-to-brain influx of endogenous and exogenous components and maintaining the homeostatic microenvironment of the central nervous system (CNS). Acute stroke leads to BBB disruption, blood substances extravasation into the brain parenchyma, and the consequence of brain edema formation with neurological impairment afterward. Caspase-1, one of the evolutionary conserved families of cysteine proteases, which is upregulated in acute stroke, mainly mediates pyroptosis and compromises BBB integrity *via* lytic cellular death and inflammatory cytokines release. Nowadays, targeting caspase-1 has been proven to be effective in decreasing the occurrence of hemorrhagic transformation (HT) and in attenuating brain edema and secondary damages during acute stroke. However, the underlying interactions among caspase-1, BBB, and stroke still remain ill-defined. Hence, in this review, we are concerned about the roles of caspase-1 activation and its associated mechanisms in stroke-induced BBB damage, aiming at providing insights into the significance of caspase-1 inhibition on stroke treatment in the near future.

## Introduction

Stroke is the second-leading cause of death and affects more than 13.7 million individuals per year worldwide^1^. About 70% of incident strokes are ischemic (9.5 million) and the rest are intracerebral hemorrhage (ICH) or subarachnoid hemorrhage (SAH) (Phipps and Cronin, 2020). Specifically, in the United States, approximately 7.9 million individuals experience a stroke, of which 87% (690,000) are ischemic, and approximately 2 million individuals experience a transient ischemic attack each year (Kleindorfer et al., 2021). Although various animal experiments for novel therapeutic drugs were promising yet, efforts were made to preserve the blood–brain barrier (BBB) integrity and minimize unfavorable outcomes in acute stroke. BBB disruption in stroke, which was critical in the pathophysiology underlying stroke and stroke-related secondary damage, draws much attention nowadays.

The BBB, a structure of tightly sealed endothelial cells (ECs) located at the luminal surface of cerebral vasculature (Sweeney et al., 2019), is composed of ECs, pericyte, astrocyte, and extracellular matrix (ECM) and is supported by neurons and vessels. Importantly, that barrier guards the central nervous system (CNS) homeostasis and regulates molecular movement between blood and brain (Begley and Brightman, 2003; Abbott et al., 2010). During acute stroke, BBB can be easily impaired *via* mechanical stretch (Liu et al., 2016a), oxidative stress (Yang et al., 2017), inflammation (Jansson et al., 2014), and metabolites (Savage et al., 2012), permitting a large inflow of hematogenous fluid containing blood-borne cells, chemicals, and fluid extravasate into brain parenchyma, giving rise to brain edema (Stokum et al., 2016; Xiao et al., 2020) and subsequent neurological impairment (Sweeney et al., 2019). Unfavorable events, such as hemorrhagic transformation (HT) (Terruso et al., 2009) and hematoma expansion (Keep et al., 2014; Sawada et al., 2017), can also be attributed to BBB dysfunction during the ischemia and hemorrhagic stroke, respectively.

Caspases are an evolutionary conserved family of cysteine proteases that are centrally involved in regulating multiple patterns of programmed cell death (Van Opdenbosch and Lamkanfi, 2019), among which apoptosis and pyroptosis contribute to BBB dysfunction the most in stroke. In contrast to the classical non-lytic apoptotic cell death, caspase-1 mainly mediates the major lytic cell death mode, namely pyroptosis, with pore formation on the cellular membrane and inflammatory cytokines release (Van Opdenbosch and Lamkanfi, 2019). Caspase-1 has been previously confirmed to mediate BBB disruption in the CNS, specifically critical in ECs’ injury and repair of the BBB (Israelov et al., 2020). As specific caspase-1 inhibitors have been confirmed to alleviate BBB deterioration since 2010 (Wu et al., 2010), targeting caspase-1 in stroke contributes to a promising end with a better functional outcome (Wu et al., 2010; Lin et al., 2018; Liang et al., 2019, 2020; Li et al., 2020b; Zhang et al., 2020), milder cerebral edema (Wu et al., 2010; Lin et al., 2018; Liang et al., 2019; Li et al., 2020b; Zhang et al., 2020), and lower occurrence of HT (Ismael et al., 2018; Chen et al., 2021a). However, the mechanisms about caspase-1 inhibition on stroke-induced BBB damage remain ill-defined yet. Thus, we will review the roles of caspase-1 on BBB in acute stroke through the priming, activation, and effects, aiming at elucidating potential therapeutic targets *via* a better understanding of the function and regulation of caspase-1 during the acute stroke.

## Caspase-1 and Caspase-1-Dependent Main Effects

**FIGURE 1:**
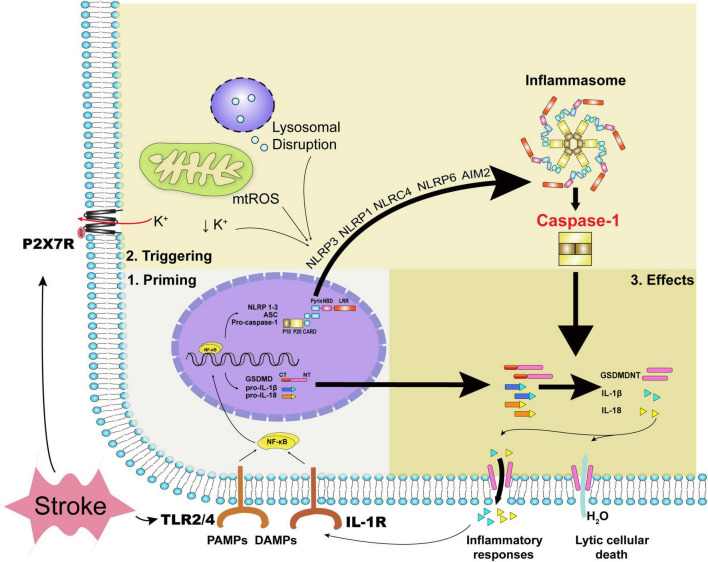
The priming process, triggering process, and executive effects of caspase-1. Acute stroke primarily leads to oxidative stress, disturbance of metabolites, and mechanical stress. DAMPs, inflammatory cytokines together with PAMPs can stimulate the priming process with the transcription of inflammasomes’ constituents and executive components such as pro-IL-1β, pro-IL-18, and GSDMD. During acute stroke, decreased intracellular potassium concentration, oxidative stress, and lysosomal disruption trigger NLRP3 inflammasome and caspase-1 activation, while dsDNA the form of cytosolic DNA activates caspase-1 *via* AIM2 inflammasome. Specifically, mechanical stress due to hematoma or edema compression can regulate ATP-gated ion channel P2X7R with potassium efflux. Caspase-1 activation leads to the formation of GSDMDNT and IL-1β or IL-18, yielding pore formation and inflammatory responses ultimately. DAMPs indicate damage-associated molecular patterns; PAMPs, pathogen associated molecular patterns; NLRP, NOD-like receptor family pyrin domain-containing; NLRC, NOD-like receptor family CARD domain containing; mtROS, mitochondrial reactive oxygen species; AIM2, absent in melanoma 2; TLR, toll-like receptor; GSDMD, gasdermin D; IL, interleukin; GSDMDNT, N-terminal fragment of gasdermin D.

Caspase-1 was the first caspase reported as a protease in 1989 (Black et al., 1989) and encoded by the sequence of 11q22.1 in humans and 9A1 in mice (McIlwain et al., 2013), widely expressed among multiple organs such as spleen, liver, intestine, and brain (Stienstra et al., 2011; Kumaresan et al., 2016; Heneka et al., 2018). Caspase-1 is generally situated in the cytosol as an inactive zymogen procaspase-1 after translation and is activated proteolytically *via* the protein complexes “inflammasomes” for proteolytic activation (Lamkanfi and Dixit, 2009). Prototypical inflammasome is a heterologous oligomeric protein complex comprising of a sensor protein from some subfamilies of pattern recognition receptors, a caspase-1 family protease, and mostly an apoptosis speck-like protein (ASC), which couples the previous two together. Various sensor proteins, comprising of several members of the intracellular nucleotide-binding domain and leucine-rich-repeat containing (NLR) family namely NLRP1b, NLRP3, NLRC4, and NLRP6, the HIN200 family member absent in melanoma 2 (AIM2), and the TRIM family member Pyrin (Van Opdenbosch and Lamkanfi, 2019), were activated *via* diverse signals and distinguished the inflammasomes from each other. The most popular one NLRP3 (also termed as cryopyrin or Nalp3) is highly expressed in astrocytes, ECs, neurons, and microglia in CNS (Cassel and Sutterwala, 2010; Liu et al., 2013). Procaspase-1 harbors an N-terminal caspase activation and recruitment domain CARD, an internal large domain (p20), and a short C-terminal domain (p10), with three domains separated by linker sequences. After inflammasome activation, procaspase-1 is transformed into caspase-1 with a tetramer linked by two symmetrically arranged p20/p10 dimers (Figure 1).

Two steps, the priming and triggering processes, were essential for caspase-1 activation, respectively. The priming process indicates the expression of inflammasome composites and inflammatory cytokines. Pathogen-associated molecular patterns (PAMPs), damage-associated molecular patterns (DAMPs), and inflammatory cytokines stimulate Toll-like receptors (TLRs), and interleukin (IL)-1R evokes nuclear factor kappa B (NF-κB) expression, along with the expression of NLRP1-3, ASC, pro-caspase-1, gasdermin D (GSDMD), pro-IL-1β, pro-IL-18, etc., subsequently (Van Opdenbosch and Lamkanfi, 2019). PAMPs refer to pathogens and related products such as lipopolysaccharide, while DAMPs refer to the immunostimulatory molecular patterns in sterile injury (Chen and Nunez, 2010). After the priming process, the assembly and triggering processes of inflammasomes subsequently proceed *via* specific cytoplasmic pattern recognition receptors, sensing that the ligands are either the same or different as the previous priming signal (Rathinam and Fitzgerald, 2016). For instance, the commonly discussed NLRP3 can be triggered mainly by mitochondrial reactive oxygen species (ROS), decreased intracellular potassium concentration, lysosomal destabilization, and the release of lysosomal cathepsins (Kelley et al., 2019). AIM2 can be triggered by double-strain DNA (dsDNA) released from dead cells (Li et al., 2020c). The triggering signals for NLRP1b, NLRC4, and NLRP6 inflammasomes in sterile inflammation during acute stroke were possible exogenous and endogenous substances, which were not clearly defined (Abulafia et al., 2009; Poh et al., 2019; Zhang et al., 2020). Apoptotic pathway can also interact with caspase-1 as the apoptotic effector caspase-8 has been found to associate with the NLRP3 inflammasome and caspase-1 activation (Sarhan et al., 2018).

After inflammasomes and caspase-1 activation, caspase-1-mediated pyroptosis ensues with abrupt cellular death and inflammatory cytokines release. Pyroptosis occurs mostly in phagocytes, such as microglial cells, but also in neurons, astrocytes, and ECs in the CNS. Lytic cell death is accomplished *via* the pore-forming N-terminal fragment of GSDMD (GSDMDNT), which is usually linked to a carboxy-terminal inhibitory domain (Liu et al., 2016b; Sborgi et al., 2016) that is kept in an autoinhibitory state and released from GSDMD *via* caspase-1-mediated cleavage (Ding et al., 2016). With the size of cellular pore approximately 10–20 nm (Ding et al., 2016; Sborgi et al., 2016), water is permeable and culminates in cell swelling and lysis (Evavold et al., 2018). Unlike the explosive rupture in necrosis, morphology underlying pyroptosis manifests as cellular swelling, flattening of the cytoplasm due to plasma membrane leakage, and lots of bubble-like protrusions appearing on the surface of the cellular membrane before its rupture (Chen et al., 2016). Moreover, increased permeability can contribute to the flux of ions with a decreased intracellular potassium concentration, further triggering canonical NLRP3 inflammasome and caspase-1 activation (Ruhl and Broz, 2015). Caspase-1 activation can increase the cleavage of multiple enzymatic substrates, including not only the classical cytokines IL-1β and IL-18, but also cytokines IL-1F7b and IL-33, a plasma membrane Ca^2+^-ATPase (PMCA2), and calpastatin, the endogenous calpain inhibitor (Winkler and Rosen-Wolff, 2015). As with other cytoplasmic molecules such as intracellular potassium and DAMPs, especially HMGB1 and ATP (Sun et al., 2020), the mature IL-1β and IL-18, which have a diameter of 4.5 nm (Ding et al., 2016) and secreted in glial cells as well as ECs, are secreted into extracellular spaces depending upon GSDMD-dependent membrane permeability and initiate inflammatory process subsequently (Tapia et al., 2019). In particular, the release of DAMPs can contribute to caspase-1 activation in a vicious cycle (Savage et al., 2012). It is noteworthy to mention that non-canonical inflammasome pathway can also be activated in the stimulus of PAMPs, mediating GSDMD-dependent membrane pore formation and intracellular potassium efflux *via* caspase-4/5 (caspase-11 in mice) without the cleavage of pro-IL-1β/pro-IL-18 (Van Opdenbosch and Lamkanfi, 2019). Moreover, IL-1β release can also be facilitated *via* the absence of pyroptosis (Evavold et al., 2018) independently of plasma membrane pore formation (Karmakar et al., 2020).

## The Activation of Caspase-1 in Acute Stroke

The blood–brain barrier can be impaired *via* mechanical stretching (Liu et al., 2016a), oxidative stress (Yang et al., 2017), inflammation (Jansson et al., 2014), and metabolites (Savage et al., 2012) in both ischemic stroke (Wu et al., 2016; Hirsch et al., 2021) and hemorrhagic stroke (Wu et al., 2010; Gan et al., 2021). Interestingly, caspase-1 can mediate those processes to some extent by driving detrimental effects on BBB in acute stroke. Therefore, we will first review the priming and triggering processes of caspase-1 during a stroke in this part.

### The Activation of Caspase-1 in Ischemic Stroke

Although various models have been developed for ischemic stroke, such as transient middle cerebral artery occlusion (MCAO; Li et al., 2019, 2020c), permanent MCAO (Dong et al., 2013; Liang et al., 2020), embolic MCAO (Ishrat et al., 2015), and photothrombotic stroke (Li et al., 2020b) etc., caspase-1 expression can be enhanced in the acute phase of all models above. For 2-h transient MCAO in mice, NLRP3 and caspase-1 expression are significantly upregulated at 12 h, peaking at 24 h, and remaining elevated for more than 48 h in a time-dependent manner (Yang et al., 2014); while for permanent MCAO in mice, NLRP3 and caspase-1 expression are detected in ischemic penumbra within 24 h, peaking at 3 days and remaining elevated for 7 days after stroke (Barrington et al., 2017; Liang et al., 2020). Two successive phases, ischemia and reperfusion, probably occur during ischemic stroke. In the ischemic phase, cerebral blood flow is reduced, resulting in a deficient supply of glucose and oxygen (Sandoval and Witt, 2008). As glucose and oxygen are essential to maintain an adequate supply of ATP to guarantee physiological cellular function and maintain normal ion gradients (Abdullahi et al., 2018), oxidative phosphorylation is discontinued (Hata et al., 2000), and ion transporters on ECs are unable to function with maintaining Na^+^–K^+^–ATPase and Ca^2+^–ATPase due to lack of energy. Thus, in the ischemic phase, decreased intracellular K^+^ concentration together with calcium overload (Abdullahi et al., 2018) mainly elicit the triggering process *via* metabolites dysregulation. When the reperfusion phase follows, blood flow starts to recover, potentially beneficial for neuronal survival, but oxidative stress can ensue first with NLRP3 and caspase-1 activation (Ishrat et al., 2015). DsDNA released from dead cells can drive inflammasomes AIM2 activation (Li et al., 2020c). Due to increased permeability, ECM degeneration, and angiogenesis mediated by BBB dysfunction (Sandoval and Witt, 2008), vasogenic and inflammatory edema formed contribute to mechanical strains and modulate triggering of NLRP3 via P2X7 receptors (Albalawi et al., 2017). Other inflammasomes such as NLRP1 (Fann et al., 2013), NLRC4 (Poh et al., 2019), and NLRP6 (Zhang et al., 2020) have also been implicated in the response of acute stroke with the triggering process shown in Figure 1.

### The Activation of Caspase-1 in Hemorrhagic Stroke

For hemorrhagic stroke, NLRP3 upregulation, caspase-1 activation, and IL-1β release occur as early as 3 h post-ICH and intensify gradually in the area around the hematoma from 1 to 5 days (Cheng et al., 2017; Yao et al., 2017). Similarly, two phases occur after a hemorrhagic stroke, as (1) the initial mechanical compression damage induced by hematomas and (2) the secondary injury characterized by excitotoxicity, inflammation, and oxidative stress.

During the initial compression by hematoma, mechanical strain-induced P2X7 receptors can activate NLRP3 inflammasomes and caspase-1 (Albalawi et al., 2017). Ischemic events can also be prevalent among hemorrhagic stroke (Prabhakaran et al., 2010; Topkoru et al., 2017), with a similar caspase-1 activation process as described above. Moreover, the breakdown of blood products was more unique in ICH. A large amount of ATP released by RBC lysis also triggered the P2X7R/NLRP3 inflammasome activation, as previously discussed (Chen et al., 2013; Feng et al., 2015). Products of hematoma, such as hemolysate degradation (hemoglobin, iron, or heme), erythrocyte thrombin, and fibrinogen, enable the priming and triggering pathways (Keep et al., 2014; Tso and Macdonald, 2014). Hemoglobin activates the TLR2/TLR4 heterodimer and promotes oxidative stress (Urday et al., 2015). Similarly, heme (Dutra et al., 2014) and iron (Mori et al., 2001) can both generate mitochondrial ROS, activate NLRP3/caspase-1 pathway, and lead to BBB hyperpermeability and cerebral edema, specifically on ECs, which can be alleviated by iron chelator deferoxamine (Lee et al., 2010). Other products, such as fibrinogen as DAMPs (Rosin and Okusa, 2011) and thrombin as the ROS contributor (Ye et al., 2017), also promote caspase-1 activation in hemorrhagic stroke.

### Other Potential Factors Related to Caspase-1 Activation Process

Meanwhile, despite the direct activation of caspase-1, several comorbidities along with stroke can exacerbate caspase-1 activation toward unfavorable outcomes. Hyperglycemia or diabetics has shown the most eminent effects on stroke so far. Diabetes can amplify NLRP3 and caspase-1 activation in rats after ischemia (Ward et al., 2019), which can mediate the facilitation of tPA-induced BBB damage (Ismael et al., 2020, 2021; Chen et al., 2021a) and the tendency of HT in ischemic stroke (Ismael et al., 2020). Hyperglycemia induces superimposed effects on caspase-1 in stroke, as specific NLRP3 inhibitor MCC950 showed stronger alleviation of BBB destructions in hyperglycemic stroke than stroke without hyperglycemia (Hong et al., 2018). Whether superimposed effects exist among caspase-1 and other risk factors on BBB disruption in stroke, such as hypertension, hyperlipidemia, as well as aging (Jiang et al., 2018), deserves further research.

## Caspase-1-Dependent Effects on BBB Dysfunction in Acute Stroke

**FIGURE 2:**
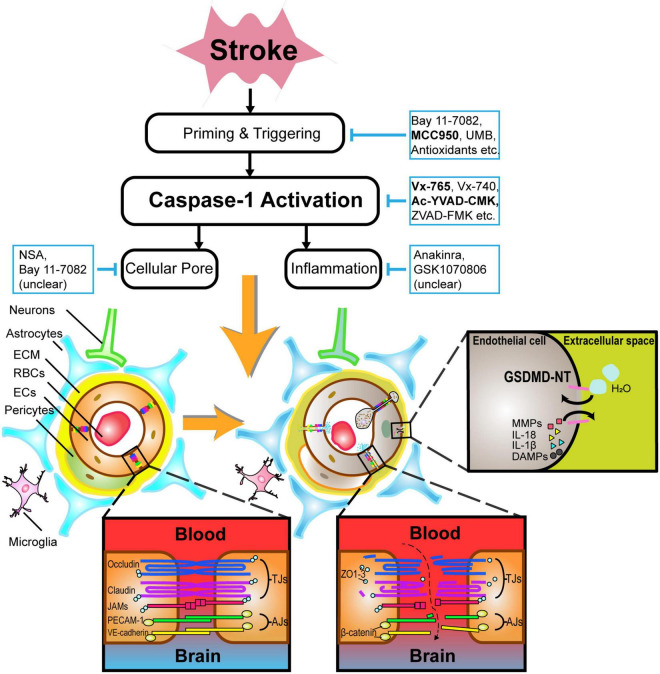
An illustration of the pathophysiology underlying caspase-1-mediated blood–brain barrier injuries and potential drugs targeting caspase-1-associated pathways in acute stroke. After caspase-1 activation, not only BBB components such as endothelial cells and astrocytes undergo lytic cellular death, but BBB supporting structures such as neurons and microglia are subject to pyroptosis likewise. In particular, the phosphorylation of tight junction proteins, particularly occludins and claudins, contribute to BBB hyperpermeability. GSDMDNT-mediated pore formation brings about the exchange of ions and water molecules together with inflammatory cytokines, cumulating in cellular swelling and lysis. Caspase-1-related neutrophils and lymphocytes transmigration also drive BBB disintegration to some extent. Alternatively, selective inhibitors of caspase-1-associated pathways such as Vx-765, Ac-YVAD-CMK, MCC950, etc., are promising in alleviating BBB injuries during acute stroke. ECM indicates extracellular matrix; RBCs, red blood cells; ECs, endothelial cells; IL, interleukin; DAMPs, damage-associated molecular patterns; MMPs, matrix metalloproteinases; GSDMDNT, N-terminal fragment of gasdermin D.

Accumulating evidence have shown caspase-1 activation plays pivotal roles in exacerbating cerebral edema (Wu et al., 2010; Lin et al., 2018; Liang et al., 2019; Li et al., 2020b; Zhang et al., 2020) and increasing the occurrence of HT (Ismael et al., 2018; Chen et al., 2021a) via loosening BBB integrity (Liang et al., 2020). Caspase-1 mainly mediates effects on pyroptosis *via* pore formation, inflammatory cytokines release and other routes such as apoptosis as well. Considering that caspase-1 can be expressed on BBB, in this part, we will focus on caspase-1-dependent effects on BBB structures during a stroke. Meanwhile, since the effects share resembling characteristics in various patterns of ischemic and hemorrhagic stroke (Wu et al., 2010; Lin et al., 2018; Li et al., 2019, 2020b; Liang et al., 2019, 2020), caspase-1-dependent effects on BBB will be concerned with stroke events as a whole (Figure 2).

### Caspase-1-Dependent Cellular Death on Blood–Brain Barrier in Acute Stroke

Caspase-1-dependent formation of plasma-membrane pores mediating lytic cell death occur mostly in glial cells, pericytes, ECs, and neurons (Zhang et al., 2016; Yang et al., 2017), as shown in Figure 2.

Microvascular ECs, which possess adherens junctions and tight junctions (TJs) proteins such as zonula occludens (ZO)-1 and occludins, are crucial for blocking the recruitment of peripheral leukocytes and preventing paracellular transport of multiple hydrophilic compounds across the BBB (Yang et al., 2013). Claudins (primarily claudin-5) and occludins are major transmembrane TJ proteins, phosphoproteins with four transmembrane domains spanning the intercellular cleft homotypically binding to proteins on adjacent ECs, and with cytoplasmic terminals binding to many cytoplasmic proteins including ZO-1, ZO-2, ZO-3, and cingulin, which are linked to actin cytoskeleton and maintain the structural and functional integrity (Stamatovic et al., 2016). Adherens junctions also have transmembrane proteins, cadherins, that bind to adjacent ECs and cytoplasmic plaque proteins β- or γ-catenin on the cytoplasmic domains forming the cadherin–catenin complex, which is also linked to actin cytoskeleton (Stamatovic et al., 2016). Pyroptosis in acute stroke can drive TJ proteins, adherens junction proteins, and actin cytoskeleton dysfunction on ECs, leading to their death eventually (Cao et al., 2016; Liang et al., 2020). Apart from structure destruction, posttranslational protein modification on TJs, namely, occludin, claudin-5, and ZO-1 can further increase BBB permeability *via* different kinases on distinct residues (Krizbai and Deli, 2003; Gonzalez-Mariscal et al., 2008) potentially *via* the Rho/Rho-associated protein kinase (Beckers et al., 2010), protein kinase C (Dorfel and Huber, 2012), and mitogen-activated protein kinase signaling pathways (Fujibe et al., 2004). Furthermore, ECs can also produce matrix metalloproteinases (MMPs), which potently degrade TJ and ECM structures after the lysis of ECs (Reuter et al., 2013).

Astrocytes belong to the neuron–glia system, and are the most abundant cell type in the CNS. Astrocytes provide many fundamental functions, including BBB formation, BBB structural support, the regulation of blood flow, etc. (Rossi, 2015), with water channel aquaporin 4 highly expressed on astrocytic endfeet, critically regulating water flux between the blood and brain (Nagelhus and Ottersen, 2013). In acute stroke, astrocytes transformed into reactive emerging roles in endogenous neuroprotection and repair (Koizumi et al., 2018). However, astrocytes still undergo the pyroptosis process (Li et al., 2020b; Zhang et al., 2020), with astrocytes swelling compressing vessels exacerbating vascular hypoperfusion (Sykova, 2001). The disruption of astrocytes together with aquaporin 4 unpolarization occurs in stroke subsequently, which can be reversed *via* targeting caspase-1, especially among the diabetics (Ward et al., 2019). Moreover, pyroptosis contributes to unregulated patterns among astrocytes and potential detrimental injury to CNS, as unregulated process induces brain edema, forms a compact glial scar, aggravates inflammation, and generates a toxic microenvironment for the components of CNS (Choudhury and Ding, 2016).

Pericytes belong to a perivascular cell type that encapsulates the microvasculature of the brain and spinal cord, forms lock and socket junctions with ECs, and contributes significantly to the maintenance of the BBB (Bennett and Kim, 2021). Pericytes can be impaired in neurovascular pathology, particularly in acute stroke (Bennett and Kim, 2021). The encapsulation rate of pericytes, which is crucial for the maintenance of microvasculature and is usually decreased during a stroke, can be elevated *via* caspase-1 inhibition (Liang et al., 2020).

Other cells, especially microglia and neurons, may also undergo pyroptosis and modulate BBB dysfunction. Microglia are mononuclear phagocytes, the main resident immune cells within the CNS, representing up to 10% of the total cell amount of the brain (Soulet and Rivest, 2008), which respond to sharp change in brain homeostasis and remove damaged cells *via* phagocytosis the first. Two distinct phenotypes of microglia have been documented (1) M1 microglia, the classically activated one, is considered to play a proinflammatory role in releasing inflammatory cytokines, including tumor necrosis factor-α, IL-1β, IL-6, IL-18, IL-23, inducible nitric oxide synthase, MMP-9, and MMP-3 (Yenari et al., 2010; Ransohoff and Brown, 2012), and (2) M2 microglia, the alternatively activated phenotype, is critical for neurogenesis, angiogenesis, and anti-inflammation by producing IL-10, growth factor including transforming growth factor β, brain-derived neurotrophic factor, and vascular endothelial growth factor (Ponomarev et al., 2013). In acute stroke, microglia show functionally distinct phenotypes according to the location and the different phases of stroke (Faustino et al., 2011). Caspase-1 activation contributes to increased classically-activated (proinflammatory) M1-type microglia polarization and reduced the number of (anti-inflammatory) M2-type cells surrounding the hematoma (Franco and Fernandez-Suarez, 2015; Hu et al., 2015; Lin et al., 2018), with a huge amount of M1 polarization induced by an increase in IL-1β secretion after hemorrhagic stroke (Zhang et al., 2017b). Similar phenomena of shifting microglia polarization can also be observed in transient ischemic mice (Li et al., 2019). Pore formation on the membrane facilitates the release of intracellular inflammatory factors from microglia, along with robust inflammation in the acute phase of ischemic stroke, leading to irreversible injury. Neurons, a crucial part in neurovascular unit supporting the BBB function, were highly susceptible to cause scattered dead neurons in the core of ischemic area (Lipton, 1999). Other than the necrotic process, pyroptosis still leads to neurons death to some degree, thereby contributing to the denervation of BBB and poor recovery of BBB (Kaplan et al., 2020). Conversely, the release cytosolic dsDNA induced by neurons leads to the triggering of AIM2 in a vicious cycle and drives a secondary immune response, including glial activation, release of cytokines and chemokines (Guruswamy and ElAli, 2017), and recruitment of peripheral immune cells (Kleinschnitz et al., 2013; Mracsko et al., 2014).

### Caspase-1-Dependent Release of Proinflammatory Cytokines on Blood–Brain Barrier in Acute Stroke

IL-1β, commonly produced by astrocytes and microglia in CNS, is one of the most extensively explored cytokines in pyroptosis during stroke (Blamire et al., 2000). Brain- and blood-derived IL-1 are both able to culminate in BBB disintegration in a transient model of focal cerebral ischemia (Denes et al., 2013) *via* the protein kinase C-theta in human brain microvascular ECs (Rigor et al., 2012). Treatment with IL-1 receptor antagonist (IL-1Ra) or overexpression of IL-1Ra using an adenoviral approach has shown a dramatical reduction of BBB injury following ischemic stroke in rodents (Betz et al., 1995; Garcia et al., 1995; Pradillo et al., 2012, 2017). The underlying pathophysiology of IL-1β on caspase-1-mediated BBB dysfunction can be as follows: (1) IL-1β directly interacts with IL-1R in microglia (Holmes et al., 2003) and upregulates the priming process; (2) IL-1 affects astrocytes and microglial functions *via* promoting the proliferation (Herx and Yong, 2001) and release of cytokines and chemokines (Parker et al., 2002; Andre et al., 2005), as well as the activation of MMP-9 (Thornton et al., 2008); (3) IL-1β can induce chemokines CXCL1 (KC), CXCL2 (MIP-2), CXCL12 (SDF-1), and adhesion molecules (Israelov et al., 2020) from astrocytes and ECs along with the release of inflammatory factors, the infiltration of leukocytes and T-cells (Blamire et al., 2000; Fogal and Hewett, 2008; Sobowale et al., 2016), and the excretion neutrophil-derived MMPs (McColl et al., 2007, 2008) to the injury site (Sedgwick et al., 2000) in Figure 2.

IL-18, originally discovered as an IFN-inducing factor, modulates IFN-production from IL-18R expressing Th1 and NK cells in the periphery (Nakanishi et al., 2001; Nakanishi, 2018). In CNS, IL-18 can be upregulated by neurons, astrocytes, and microglia following lipopolysaccharide stimulation or treatment with IFN-γ (Alboni et al., 2010). Although IL-18 was shown to mediate inflammatory response resulting in loss of appetite, sleep dysregulation, and several neurodegenerative diseases (Alboni et al., 2010), caspase-1 mediated IL-18 on BBB in stroke deserves further exploration.

### Interaction of Caspase-1 With Other Programmed Cell Death Pathways on Blood–Brain Barrier in Acute Stroke

The apoptotic pathway, which is functionally mediated by initiator (caspases 8, 9, and 10) (Behrouzifar et al., 2018; Israelov et al., 2020) and effector (caspases 3, 6, and 7) (Liu et al., 2017) caspases, can also mediate BBB dysfunction with increased hyperpermeability. Interestingly, the apoptotic pathway can partially interact with caspase-1. To some extent, caspase-8 can regulate the priming and activation of the canonical and non-canonical inflammasome pathways and modulate proIL-1β maturation (Van Opdenbosch and Lamkanfi, 2019). Caspase-1 activation has also shown the ability to induce apoptosis in GSDMD-deficient macrophages by engaging Bid-caspase-9-caspase-3 axis through the intrinsic apoptosis pathway (Tsuchiya et al., 2019). It is noteworthy mentioning that NLRP3 and AIM2 agonists can also induce caspase-8-mediated apoptosis (Pierini et al., 2012; Sagulenko et al., 2013) in caspase-1-deficient macrophages. However, fewer protective effects on BBB were found *via* caspase-8 or caspase-9 inhibition than caspase-1 inhibition (Israelov et al., 2020). Thus, targeting caspase-1 and related pathway has been suggested to be more effective in maintaining the integrity of BBB.

## Therapeutic Targets of Caspase-1 and Its Associated Pathways

Targeting caspase-1 and its associated pathways has been suggested as an efficient method to alleviate BBB damage throughout acute stroke. We will review potentially effective drugs that target caspase-1 and promote protective effects on BBB in acute stroke in the following part, as listed in Table 1.

**TABLE 1:** The drugs preserving blood-brain barrier integrity via direct caspase-1 inhibition in acute stroke.

Stroke category	Animals/cells	Outcomes *in vivo*	Inhibitors	Target of inhibitors	Protective mechanisms on BBB	References
Ischemic-HT	*In vivo* (mice)	↓ HT and edema, ↑ neurological function	MCC950, Ac-YVAD-CMK, diacerein	NLRP3, Caspase-1, IL-1β	↓ Inflammatory cytokines.	Chen et al., 2021a
Ischemia	*In vivo* (rats)	↓ Infarction volume, edema and neurodeficits	Vx-765	Caspase-1	(1) ↑ CD31, ZO-1, occludin and claudin-5 expression. (2) ↑ Encapsulation coverage of pericytes. (3) ↓ BBB permeability and ↑ BBB integrity: ↓ Evans Blue leakage, ↑ GLUT-1 and osteopontin expression. (4) ↓ MMPs and ↑ TIMPs	Liang et al., 2020
Ischemia	*In vitro* (astrocytes, OGD/R, NLRP6 overexpression)	NA	Ac-YVAD-CMK	Caspase-1	(1) ↓ NLRP6 overexpression-induced pyroptosis. (2) ↓ Inflammatory cytokines.	Zhang et al., 2020
Ischemia	*In vivo* (mice)	↓ Infarction volume and neurodeficits	Vx-765	Caspase-1	↓ Pyroptosis of astrocytes and other BBB supporting structures neurons and microglia.	Li et al., 2020b
Ischemia	*In vivo* (mice)	↓ Infarction volume and neurodeficits	Vx-765	Caspase-1	(1). ↑ Microglia polarization from M1 phenotype toward M2 phenotype. (2) ↓ Inflammatory cytokines IL-1β.	Li et al., 2019
ICH	*In vivo* (mice)	↓ Brain edema and ↑ neurological functions	Ac-YVAD-CMK	Caspase-1	(1) ↓ ZO-1 degradation. (2) ↓ MMP9. (3) ↓ Inflammatory cytokines.	Wu et al., 2010
ICH	*In vivo* (rats)/*In vitro* (microglia, thrombin)	↓ Brain edema and ↑ neurological functions	Ac-YVAD-CMK	Caspase-1	(1) ↓ Microglia activation and infiltration. (2) ↓ Inflammatory cytokines.	Liang et al., 2019
ICH	*In vivo* (mice)	↑ Neurological functions	Ac-YVAD-CMK	Caspase-1	(1) ↓ M1-type microglia activation and ↑ M2-type activation. (2) ↓ Inflammatory cytokines.	Lin et al., 2018
SAH	*In vivo* (rabbits)	↓ Vasoconstriction	Z-VAD-FMK	Pan-caspase	(1) ↓ Infiltrating leukocytes. (2) ↓ ECs secreting endothelin-1 induced by IL-1β.	Iseda et al., 2007

*BBB, blood–brain barrier; MMPs, matrix metalloproteinases; TIMPs, tissue inhibitors of metalloproteinases; HT, hemorrhagic transformation; GLUT-1, glucose transporter type 1; ICH, intracerebral hemorrhage; SAH, subarachnoid hemorrhage; ECs, endothelial cells; OGD/R, oxygen glucose deprivation/reperfusion; NLRP, NOD-like receptor family pyrin domain-containing; IL, interleukin; ZO, zonula occludens.*

### Caspase-1 Inhibitors

VX-740 (Pralnacasan) and VX-765 (Belnacasan), two analog peptidomimetic inhibitors of caspase-1 are converted rapidly to their active forms, VRT-18858 and VRT-043198 respectively, under the action of plasma and liver esterases (Rudolphi et al., 2003; Wannamaker et al., 2007). Ac-YVAD-CMK, as another specific and irreversible inhibitor of caspase-1, is a tetrapeptide sequence based on the target sequence of caspase-1 in pro-IL-1β (Garcia-Calvo et al., 1998). Except for specific caspase-1 inhibitors, pan-caspase inhibitors such as Z-VAD-FMK (Iseda et al., 2007; Lin et al., 2016) and Q-VD-OPh (Caserta et al., 2003), which inhibit the activation of some but not all caspases, have been applied to cell death researches, including pyroptosis and apoptosis. Vx-765 (Liang et al., 2020) and Ac-YVAD-CMK (Wu et al., 2010) have been commonly used in the protection of BBB in acute stroke. Liang et al. (2020) reported that Vx-765 ameliorated the BBB leakage during acute stroke with increased expression of ZO-1, occludin, and claudin-5; enhanced encapsulation rate of pericytes; downregulated proinflammatory mediators MMPs; and upregulated tissue inhibitors of metalloproteinases. Similarly, in ICH, Ac-YVAD-CMK showed similar protective effects on TJs with ZO-1 maintenance and inflammatory pathways with decreased IL-1β and MMP-9 (Wu et al., 2010). Less edema formation (Wu et al., 2010; Lin et al., 2018; Liang et al., 2019) and less occurrence of HT (Chen et al., 2021a) were common in the application of Vx-765 and Ac-YVAD-CMK (Wu et al., 2010), while upregulating the levels of endothelial markers CD31 and TJs (Liang et al., 2020) and reducing the phosphorylation of tight junction (Liang et al., 2020) were prominent with the usage of Vx-765. Lin et al. (2018) also suggested the potential role of microglia in mediating BBB dysfunction as Ac-YVAD-CMK reduces M1-type microglia polarization and increases the number of M2-type cells surrounding the hematoma.

Despite direct inhibition of caspase-1, thalidomide is an effective anti-inflammatory drug that significantly inhibits the activity of caspase-1, but its application is limited because of its strong teratogenic activity (Keller et al., 2009). Ritonavir, once a protease inhibitor used to treat HIV, was later found to effectively reduce the level of IL-18 in mouse pancreatic cancer by suppressing caspase-1 activation (Kast, 2008) with the potential application in stroke.

### Caspase-1 Upstream Pathways Inhibition

#### Inhibitors of the Priming Process

After the addition of a specific inhibitor of NF-κB (Bay 11-7082), the priming of NLRP3 inflammasome and caspase-1 was dose-dependently reduced (Bauernfeind et al., 2009). Dong et al. (2013) reported that parthenolide, *via* the downregulation NF-κB, phospho-p38 mitogen-activated protein kinase, and caspase-1 expressions, has shown the amelioration of BBB permeability and brain damage from infarction in rats with permanent MCAO. A20/tumor necrosis factor-α-induced protein 3 is a key molecule that inhibits NF-κB pathway activation by ubiquitination and exhibits significant anti-inflammatory effects (Wertz et al., 2004), and D-series of resolve in can upregulate A20 expression and inhibit NF-κB activation to produce anti-inflammatory effects and minimize the effects of NLRP3 (Voet et al., 2018; Wei et al., 2020). Dexmedetomidine, a highly selective adrenergic α2 receptor agonist through suppressing the TLR4/NF-κB pathway, can downregulate the expression levels of NLRP3, ASC, caspase-1, and IL-1β in ICH (Song and Zhang, 2019), and upregulate the expression of TJ proteins in SAH mice (Yin et al., 2018). Theaflavin has also shown anti-inflammatory effects targeting TLR4/NF-κB p65 signaling pathway to protect BBB integrity in rat ICH model (Fu et al., 2018). Moreover, directly attenuating the stability and expression levels of NLRP3 mRNA *via* conserved microRNA-223 (Yang et al., 2015), microRNA-668 inhibitors (He and Zhang, 2020), or small interfering RNA (Yao et al., 2017) can also acquire similar effects on decreasing NLRP3 expression.

#### Inhibitors of the Triggering Process

Although the inhibition of NLRP3 (Chen et al., 2021a), NLRC4 (Gan et al., 2021), AIM2 (Li et al., 2020c), or NLRP6 (Meng et al., 2019) inflammasomes could all show the protective roles on BBB in acute stroke, multiple evidence have focused on NLRP3 due to its association with endogenous stimulus (Cao et al., 2016; Ward et al., 2019; Palomino-Antolin et al., 2021). MCC950 (also known as CP-456773 and CRID3) is the selective inhibitor of NLRP3 inflammasome by blocking ATP hydrolysis of NLRP3 by directly binding to the NACHT domain (Ren et al., 2018; Coll et al., 2019). Ward et al. (2019) confirmed that MCC950 maintained the vascular integrity, attenuated brain edema, improved neurological outcome and cognitive function after stroke. Blockade of NLRP3 via MCC950 in both early-phase and post-reperfusion phase among transient ischemic models protects the mice from I/R injury by mitigating inflammation and stabilizing the BBB, particularly in a dose-dependent manner (Franke et al., 2021; Palomino-Antolin et al., 2021). Similar protective effects of MCC950 on BBB have also been documented by Ren et al. (2018) in ICH.

As discussed previously, targeting NLRP3 inflammasomes that activate the process *via* decreased K^+^, mitochondrial ROS, and lysosomal disruption can also be effective in maintaining BBB structure. In ICH rats, blocking P2X7R *via* small interfering RNA, which can be a promising therapeutic method, can minimize the efflux of potassium concentration, attenuate inflammatory progression and brain damage *via* downregulating mechanical effects on NLRP3 (Feng et al., 2015). Thioredoxin-interacting protein (TXNIP), which is required for the activation of NLRP3 inflammasome (Zhou et al., 2010; Ishrat et al., 2015), is an endogenous inhibitor of the thioredoxin system, a major cellular thiol-reducing and antioxidant system (Watanabe et al., 2010). Umbelliferone (Wang et al., 2015), ruscogenin (Cao et al., 2016), ketogenic diets (Guo et al., 2018), and EGb761 (Du et al., 2020) treatment also suppressed NLRP3 inflammasome activation *via* attenuating the TXNIP/NLRP3 pathway and potentially upregulating the expression of TJ proteins (Cao et al., 2016). Through suppressing the endoplasmic reticulum AMPK/TXNIP/NLRP3 signaling pathway, Xu et al. (2019b) reported that exogenous apelin-13 significantly preserved BBB integrity, attenuated brain edema, and improved long-term spatial learning and memory abilities after SAH. The activation of peroxisome proliferator-activated γ receptor, such as pioglitazone (Xia et al., 2018) and medioresinol (Wang et al., 2021b), has less mitochondrial ROS production on brain microvascular ECs (Wang et al., 2021b). Other antioxidants which inhibit oxidative stress-induced NLRP3 via NADPH are NOX inhibitor apocynin (Qin et al., 2017), mPTP inhibitor (TRO19622) and mitochondrial ROS scavenger (Mito-TEMPO)(Ma et al., 2014), Nrf2 pathway promotor isoliquiritigenin (Zeng et al., 2017), and hydrogen inhalation (Zhuang et al., 2019) or hydrogen-rich saline (Shao et al., 2016), all preserve BBB function, in particular ECs (Zhuang et al., 2019) during acute stroke. As spleen tyrosine kinase-dependent ROS production can similarly activate NLRP3 inflammasomes, the facts that TREM-1 facilitates the recruitment of spleen tyrosine kinase and microglial pyroptosis can be blocked by specific TREM-1 inhibitor LP17 in experimental SAH (Xu et al., 2021a). Meanwhile, NEK7 was an essential protein that acts downstream of potassium efflux to mediate NLRP3 inflammasome assembly and activation (He et al., 2016), and NEK7 small interfering RNA injection also reversed BBB opening and microglia accumulation in SAH mice (Li et al., 2020a). Other non-specific drugs such as minocycline (Li et al., 2016; Lu et al., 2016), melatonin (Dong et al., 2016), resveratrol (Zhang et al., 2017a), chrysophanol (Zhang et al., 2014), glibenclamide (Xu et al., 2019a), and sinomenine (Qiu et al., 2016) have been reported to alleviate cerebral edema or early brain injury upon modulating the NLRP3 inflammasomes. Moreover, Zhang et al. (2019) reported that as a lysosomal stabilizing agent, dexamethasone and TAK1 inhibitor 5Z-7-oxozeaenol (Xu et al., 2021b), can prevent lysosomal membrane permeability and caspase-1 activation in ECs during nicotine-induced ECs disruption, despite not validated on the cerebral vasculature yet.

Inhibition of other canonical inflammasomes such as AIM2 (Li et al., 2020c), NLRP6 (Meng et al., 2019), or NLRC4 (Gan et al., 2021), can potentially preserve BBB in acute stroke as well. The reduction of AIM2 inflammasome and caspase-1 expression *via* cyclic GMP–AMP synthase antagonist A151 (Li et al., 2020c), NLRP6 reduction *via* NLRP6 siRNA (Meng et al., 2019), and restraining NLRC4 activation *via* RGS2 (Gan et al., 2021) all show the attenuation of cerebral edema.

### Caspase-1 Downstream Pathways Inhibition

#### Gasdermin D Inhibitors

Necrosulfonamide has also been identified as a chemical inhibitor of GSDMD, which directly bonds with GSDMD and inhibits GSDMDNT oligomerization (Rathkey et al., 2018). Bay 11-7082, a previously identified NF-κB inhibitor, has been reported to potently inhibit pyroptosis through interfering GSDMD pore formation and IL-1β secretion, with its extensive suppression of inflammasomes priming, specks formation as well as inflammatory caspases activation (Wang et al., 2021a). Due to the lack of specificity, the toxicity of Bay 11-7082 in cells and tissue causes its restricted application (Nandhu et al., 2017). Disulfiram is also a GSDMD inhibitor, covalently modifying cysteine 191/192 in GSDMD while not interfering with other members of gasdermin family, which abrogates the process of pore formation with normal intervening IL-1β and GSDMD processing (Hu et al., 2020). Nevertheless, little evidence was about its protective effects on BBB.

#### Proinflammatory Biomarkers Inhibitors

The specific inhibitors of IL-1β, such as diacerein, have also been proven to alleviate brain edema, minimize HT, and improve neurological outcome in ischemic stroke rats (Chen et al., 2021a). Anakinra, a recombinant IL-1Ra, prevents IL-1 signaling with the validation of its role in chronic inflammatory disease (Cavalli and Dinarello, 2018) and the risk of serious infection (Galloway et al., 2011). Canakinumab is also a fully human monoclonal anti-IL-1β antibody directed against human IL-1β. Alternatively, GSK1070806, a neutralizing humanized monoclonal antibody, and Tadekinig alfa, a recombinant human IL-18 binding protein, are both involved in capturing bioactive IL-18 away from its receptor. The roles of monoclonal antibodies so far mainly focused on neutralizing proinflammatory cytokines in autoinflammatory diseases or potentially life-threatening infections (Van Opdenbosch and Lamkanfi, 2019); however, the values of monoclonal antibodies for cytokines on BBB protection in acute stroke also deserve further evaluation.

### Non-specific Therapeutic Targets

Moreover, there were some therapeutic methods with less specific target but can interfere with caspase-1 in BBB protection. Hypothermia could reverse the expression NLRP3, cleaved caspase-1, and GSDMDNT together with increased autophagy in diabetic rats with permanent MCAO (Tu et al., 2019), and rescue the BBB biomarkers such as the increment of ZO-1 and claudin-5 (Tu et al., 2019). Chen et al. (2021b) reported that atorvastatin markedly increased survival rate, attenuated brain water content, downregulated the protein expression of NLRP1, cleaved caspase-1, IL-1β, and IL-18 with less early brain injury in SAH. Other promising drugs, such as HSYA together with Lex (Tan et al., 2020), etc., are also capable of decreasing cerebral edema *via* suppressing pyroptosis process to some degree.

## Prospectives and Conclusion

Caspase-1 can be primed, triggered, and activated during acute stroke, and can participate in BBB dysfunction due to stroke-related oxidative stress, metabolic dysfunction, and mechanical stress. Targeting caspase-1 is effective in maintaining BBB integrity as less brain swelling, fewer occurrences of HT, and better functional recovery. Considering the caspase-1 activation to be a hub, which is converged by various triggering pathways to promote diverse programmed death patterns, namely, pyroptosis and apoptosis, it should be notably indispensable and potent during a stroke. In detail, the underlying pathophysiology about BBB impairment in stroke such as protein phosphorylation, breakdown of tight junction proteins, lytic cellular death upon BBB, and supporting structures as well as neutrophils and lymphocytes transcytosis, all can be reversed by selective caspase-1 inhibition in some extent. Our review sheds light on caspase-1 as a promising and crucial target throughout stroke intervention in the coming future.

So far, caspase-1 knock-out mice appear fertile and healthy (Kuida et al., 1995). In the clinical trials, Vx-765 is also safe in resistant-epilepsy treatment, but less effective in resistant-epilepsy treatment during a Phase IIb study, while Vx-740 is safe and significantly reducing joint symptoms and inflammation in patients with rheumatoid arthritis in Phase I/IIa studies but confirmed potential liver toxicity after long-term administration in animal studies (Kudelova et al., 2015). Thus, targeting caspase-1 should be safe and practical in humans. Although stroke and stroke-related comorbidities can lead to enhanced caspase-1 activity, there is little clinical evidence about targeting caspase-1 in stroke or stroke-associated comorbidities nowadays. Future researches about caspase-1 on BBB dysfunction in stroke will progress on, including (1) the interactions of caspase-1 with other programmed death pathways such as ferroptosis potentially linked by oxidative stress, (2) more prompt targets along the pathways of caspase-1-mediated BBB dysfunction, (3) the roles of targeting caspase-1 in hematoma expansion given that hematoma expansion may associate with BBB dysfunction, (4) clinical application of caspase-1 as biomarkers in predicting adverse events such as HT, and (5) the time-window, effectiveness, and dosage of drugs targeting caspase-1 in clinical practice.

## Author Contributions

LL and SZ led and conceptualized the study. XY, GS, QL, and YF searched for documents about this topic. XY and SH reviewed and sorted those collected documents. XY and LL wrote the first draft. SZ reviewed and edited the final draft. All authors contributed to the article and approved the submitted version.

## Conflict of Interest

The authors declare that the research was conducted in the absence of any commercial or financial relationships that could be construed as a potential conflict of interest.

## Publisher’s Note

All claims expressed in this article are solely those of the authors and do not necessarily represent those of their affiliated organizations, or those of the publisher, the editors and the reviewers. Any product that may be evaluated in this article, or claim that may be made by its manufacturer, is not guaranteed or endorsed by the publisher.

## Footnotes

1
http://world-stroke.org

## Abbreviations

BBB: blood–brain barrier
ECs: endothelial cells
ECM: extracellular matrix
CNS: central nervous system
HT: hemorrhagic transformation
ASC: apoptosis-associated speck-like protein containing a caspase recruitment domain
NLR: nucleotide-binding domain leucine-rich repeat containing gene family
AIM2: absent in melanoma 2
PAMPs: pathogen-associated molecular patterns
DAMPs: damage-associated molecular patterns
TLRs: toll-like receptors
IL: interleukin
NF- κ B: nuclear factor kappa B
GSDMD: gasdermin D
GSDMDNT: N-terminal fragment of GSDMD
ROS: reactive oxygen species
MCAO: middle cerebral artery occlusion
dsDNA: double-strain DNA
TJs: tight junctions
ZO: zonula occludens
ROCK: Rho/Rho-associated protein kinase
MMPs: matrix metalloproteases
ICH: intracerebral hemorrhage
SAH: subarachnoid hemorrhage
TXNIP: thioredoxin-interacting protein
IL-1Ra: IL-1 receptor antagonist.
